# A Continuum Robot and Control Interface for Surgical Assist in Fetoscopic Interventions

**DOI:** 10.1109/LRA.2017.2679902

**Published:** 2017-03-08

**Authors:** George Dwyer, Francois Chadebecq, Marcel Tella Amo, Christos Bergeles, Efthymios Maneas, Vijay Pawar, Emanuel Vander Poorten, Jan Deprest, Sebastien Ourselin, Paolo De Coppi, Tom Vercauteren, Danail Stoyanov

**Affiliations:** Surgical Robot Vision Group, Centre for Medical Image Computing, University College London, London WC1E 6BT, U.K.; Surgical Robot Vision Group, Centre for Medical Image Computing, University College London, London WC1E 6BT, U.K.; Translational Imaging Group, Centre for Medical Image Computing, University College London, London WC1E 6BT, U.K.; Translational Imaging Group, Centre for Medical Image Computing, University College London, London WC1E 6BT, U.K.; Translational Imaging Group, Centre for Medical Image Computing, University College London, London WC1E 6BT, U.K.; TouchLab, University College London, London WC1E 6BT, U.K.; Department of Mechanical Engineering, Katholieke Universiteit Leuven, Leuven 3000, Belgium; Department of Obstetrics and Gynaecology, University Hospital Leuven, Leuven 3000 Belgium, and also with the Department of Obstetrics and Gynaecology, University College London, London WC1E 6BT, U.K.; Translational Imaging Group, Centre for Medical Image Computing, University College London, London WC1E 6BT, U.K.; Institute of Child Health, University College London, London WC1E 6BT, U.K.; Translational Imaging Group, Centre for Medical Image Computing, University College London, London WC1E 6BT, U.K.; Surgical Robot Vision Group, Centre for Medical Image Computing, University College London, London WC1E 6BT, U.K.

**Keywords:** Surgical robotics, laparoscopy medical robots and systems flexible robots mechanism design

## Abstract

Twin–twin transfusion syndrome requires interventional treatment using a fetoscopically introduced laser to sever the shared blood supply between the fetuses. This is a delicate procedure relying on small instrumentation with limited articulation to guide the laser tip and a narrow field of view to visualize all relevant vascular connections. In this letter, we report on a mechatronic design for a comanipulated instrument that combines concentric tube actuation to a larger manipulator constrained by a remote centre of motion. A stereoscopic camera is mounted at the distal tip and used for imaging. Our mechanism provides enhanced dexterity and stability of the imaging device. We demonstrate that the imaging system can be used for computing geometry and enhancing the view at the operating site. Results using electromagnetic sensors for verification and comparison to visual odometry from the distal sensor show that our system is promising and can be developed further for multiple clinical needs in fetoscopic procedures.

## I Introduction

**Fig. 1:**
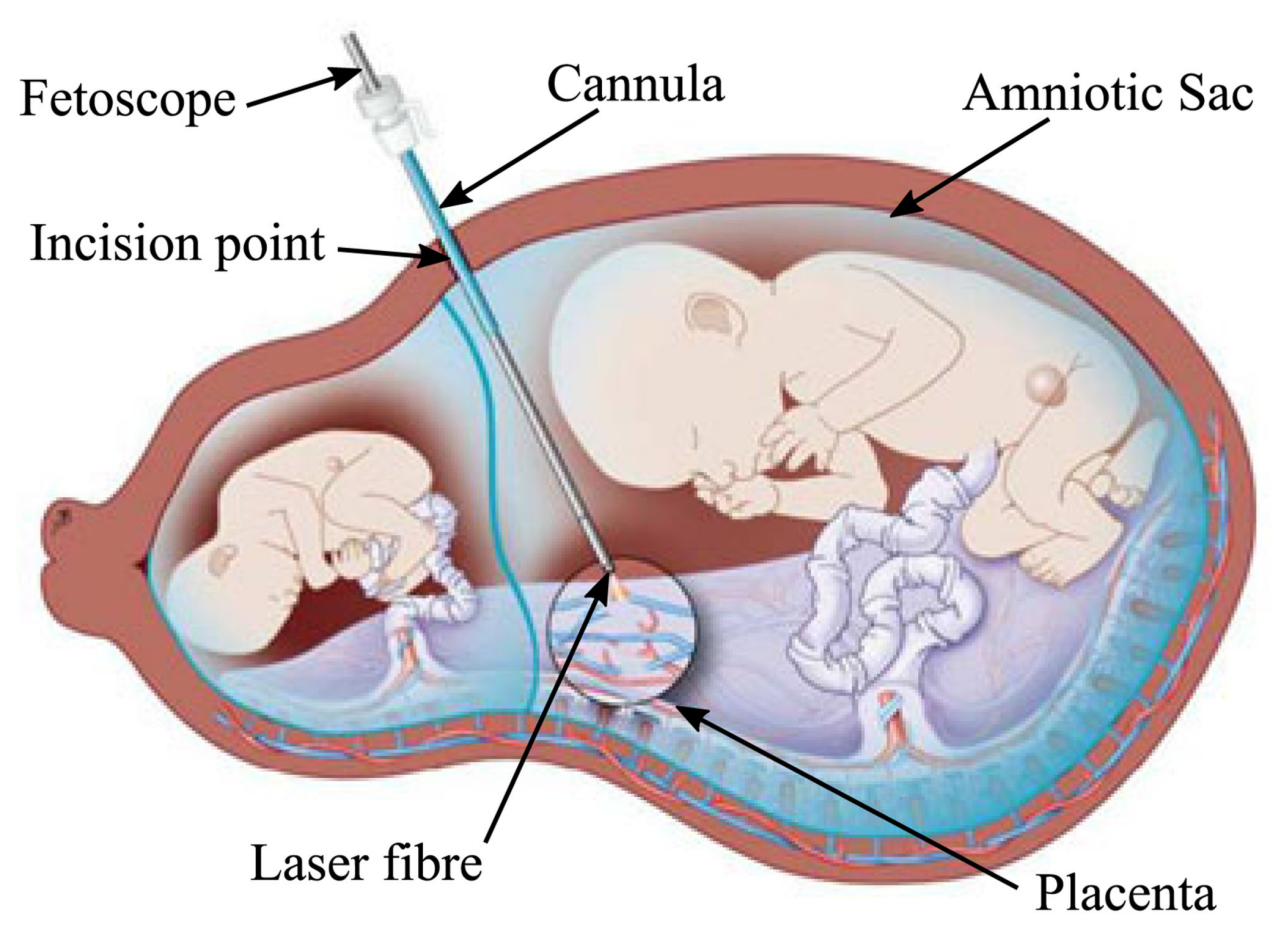
Schematic diagram of the FLP procedure for the treatment of TTTS showing with the endoscope positioned to coagulate the placental vessel anastomosis, Image reproduced with permission from UZ Leuven, Belgium.

**Fig. 2:**
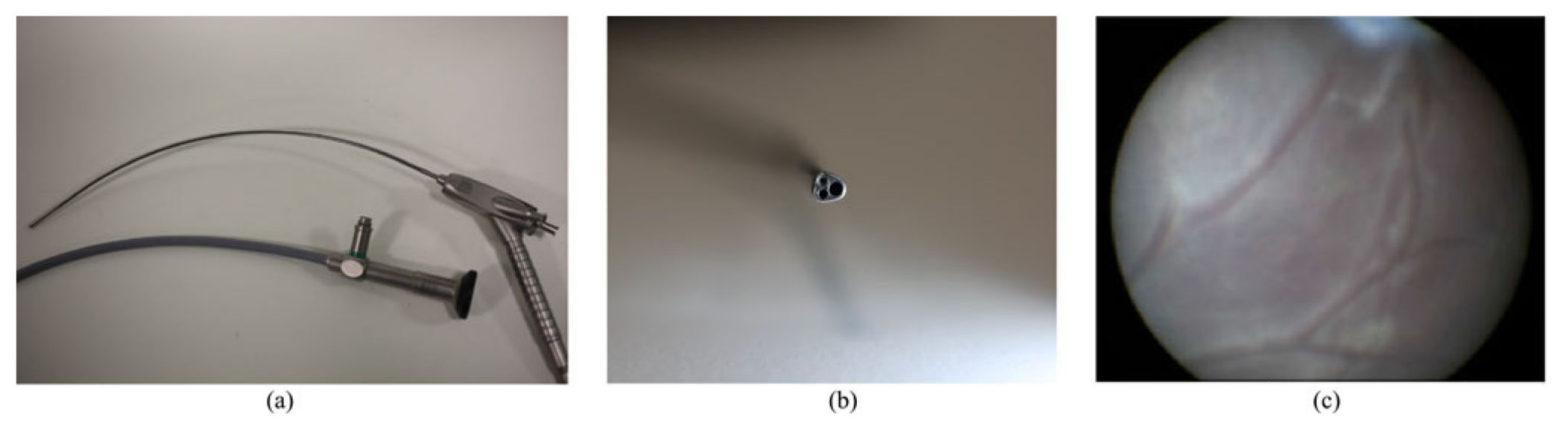
(a) Example of current fetoscopes (Karl Storz, DE), the curved shaft allows the visualisation of the placenta when it is positioned on the anterior (abdomen side); (b) Tip of the fetoscope, with optical, lighting and working channels; (c) typical image observed from the fetoscope showing a limited field of view of the internal placental wall.

Fetoscopic Laser Photocoagulation (FLP) is a minimally invasive fetal intervention used to treat Twin-Twin Transfusion Syndrome (TTTS) [1]. TTTS is caused by inter-twin vascular anastomoses on a monochorionic placenta, a shared blood supply between fetuses joined to the same placenta, causing an imbalance in the blood flow, with one fetus receiving too much blood and the other too little. While this condition is rare, it occurs in approximately 20% of twins sharing a placenta which make up 0.3% of pregnancies and it can result in the death or severe impairments of both fetuses [2]. FLP is used to coagulate the vessels (Fig. 1), thus severing the link, by using a laser fibre through the working channel of a fetoscope (Fig. 2). During FLP, the surgeon uses a rigid (straight or curved depending on the location of the placenta) fetoscope in order to observe the placenta and selectively coagulate vessels after visually determining the extent of the shared blood supply. The fetoscope is inserted into the amniotic sac through a keyhole incision passing through the abdomen, uterus and amniotic membranes, which constrains its motion and limits the areas that can be treated. An additional challenge is that forces exerted at the entry port can cause weakening of the fetal membranes and should therefore be minimised [3]. Additionally, the surgeon must refrain from physical contact with the placenta which can cause bleeding and lead to a loss of sight and complications [1]. Therefore maintaining the fetoscope at an approximately even distance from the tissue to deliver appropriate laser power is challenging and not always possible depending on the position of the placenta. In current procedures, up to 30% of shared vessels are missed or not fully coagulated [1].

To address the challenges of FLP it is possible to increase the dexterity and stability of the fetoscope by introducing actuated components to the fetoscope design. Greater dexterity of the tip of the instrument can facilitate observing and delivering therapy to the anterior placenta [4] while stability can be controlled by an articulated arm which constrains movement around a RCM at the incision point [5]. A major challenge for delivering robotic actuation to fetoscopic instrumentation is size. Continuum mechanisms have been used in surgical robotics to facilitate smaller diameter instruments and while increasing the number of degrees-of-freedom (DOF) [6], [7]. However, these mechanisms are often applied to single port surgery, intravascular or neurosurgery, where the mechanism is fixed outside the body (proximal to the surgeon) and the mechanism controls only the movement from within (distal to the surgeon). An example of continuum manipulators would be concentric tube robots [8], [9]. This manipulator category uses a series of precurved tubes positioned concentrically, the overall shape of the tubes can be controlled by translating and rotating each tube. Concentric tubes have been demonstrated with over six DOF, though they often have a comparatively low position accuracy (3–4 mm) without the use of external sensors [10], [11]. However, as the overall shape can be controlled through proximal motion, this allows for dense path planning [12] and other control schemes such as “follow the leader” deployment [13] to be implemented. In comparison, more established articulated mechanisms such as those used in the da Vinci surgical robot (Intuitive Surgical, US) utilise both proximal and distal motion from the parallel linkage and wrist joints separately. This approach results in seven active DOF, four proximal and three distal (one of which being the end effector). Instruments featuring continuum mechanisms with both distal and proximal motion have previously been presented using concentric tube robots coupled with a passive proximal arm for single port prostate surgery [14]. The concentric tube robot was inserted through an endoscope with a working channel allowing the endoscope to be manipulated with the passive arm and the robot to be manipulated relative to the endoscope. However, the proximal arm was mainly used for the initial positioning of continuum mechanism and the comanipulation of the device was not fully explored. Additionally, a concentric tube mechanism has been integrated into a da Vinci instrument providing three distal DOF along with the da Vinci three proximal DOF constrained to a mechanical RCM [15].

In this letter, we present a mechanism design and control setup of a 2DOF concentric tube robot coupled to a 7DOF robotic arm constrained to a RCM. The combination of the two actuation mechanisms allows the end-effector to be positioned with 5DOF. The mechanism was designed to be compact and attachable to the proximal robotic arm. A stereoscopic micro CMOS camera is also integrated into the tip of the inner tube. The workspace, accuracy and repeatability of the coupled mechanism is then demonstrated by tracking the tip of the nitinol tube using electromagnetic sensors as well as through visual odometry. To our knowledge this is the first time a concentric tube mechanism has been coupled to a redundant proximal arm and constrained to a virtual RCM.

## II Instrument Design

Our instrument is designed to assist in the imaging of the placenta through the inclusion of distal manipulation allowing the orientation of the tip to be decoupled from the position of the instrument shaft. For FLP, there are two main considerations in the design of each tube. For the inner tube it is the arc angle of the curved section and the bending radius. The arc angle determines the angular offset between the shaft axis and the tip axis, while the bending radius is an important consideration for imaging devices (particularly fibre based devices) as they often have a restricted bending radius. For the outer tube it is the outer diameter and the length. Current fetoscopes have an outer diameter ≤ 3.0 mm depending on the inclusion and size of the working channel and a length between 200 and 300 mm. However, a previously developed endoscope designed for FLP had a length of 150 mm [16]. These values will be used as size constraints for the instrument design.

The distal actuation mechanism uses two tubes: an outer steel tube is used as the instrument shaft and fixed relative to the proximal manipulatorand an inner curved nitinol tube positioned concentrically to the outer tube. The curved tube can be translated and rotated along the outer tube axis. One of the main advantages of using a concentric tube robot for FLP applications is that a working channel is inherently available. Therefore, there is an open centred channel allowing curvature restricted imaging and therapeutic components to be integrated within the system. Our distal mechanism is designed to allow continuous rotation of the tube to prevent any mechanical restrictions on the manipulation capabilities, to be easily attached to the proximal actuation mechanism, and for the mechanism to be compact and lightweight.

**Fig. 3:**
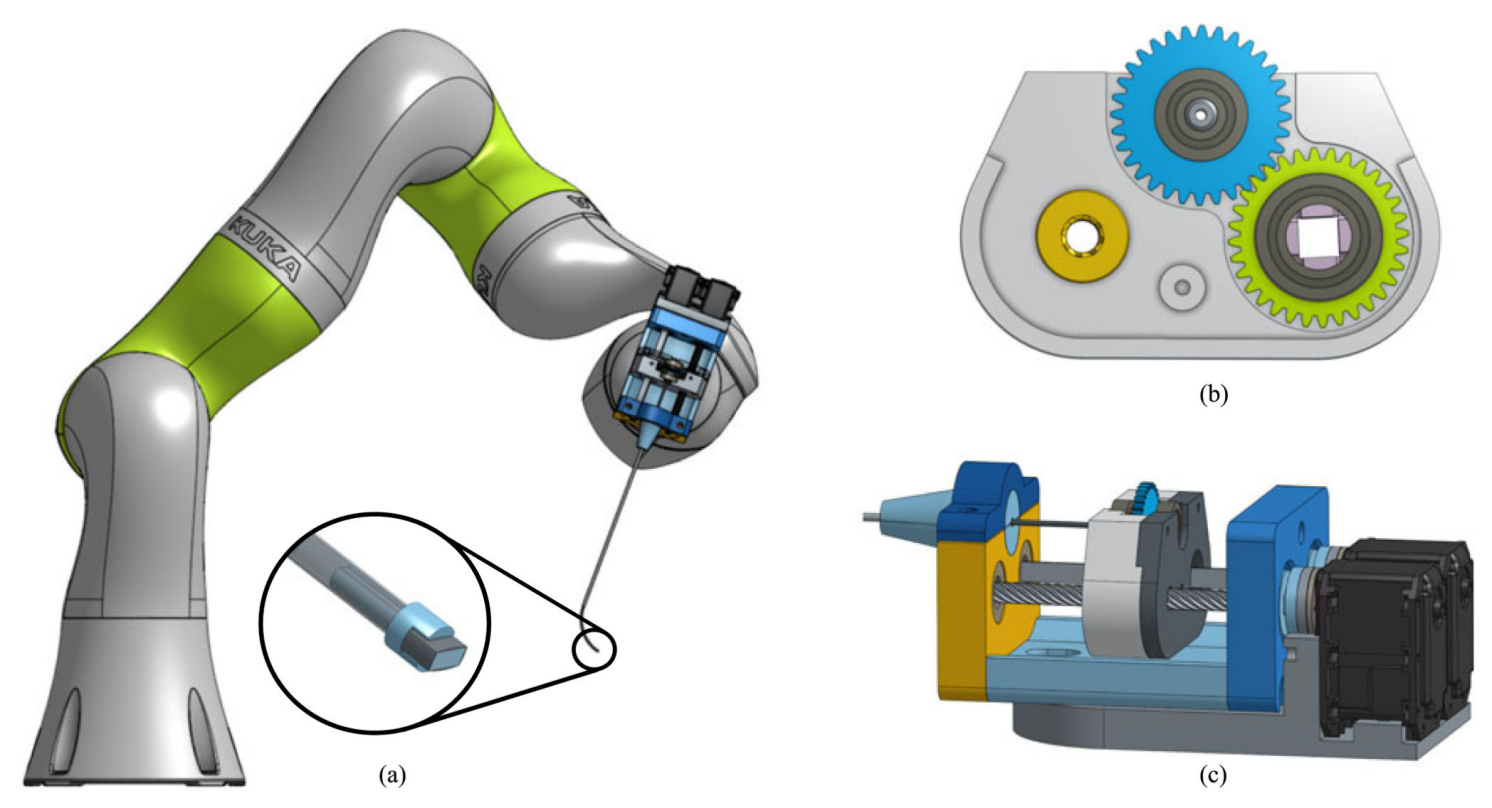
Diagram of the mechanism coupled to the KUKA LBR iiwa 7 R800; a) zoomed view of the tip and camera mount; b) Diagram of the carriage with: the lead screw nut (yellow); tube holder with the bearings and gear (blue); and rotation component with the square shaft couple, bearings and gear (green); c) View of the entire concentric tube mechanism.

Our design focuses on the use of a carriage as an interface to transfer torque from motors, attached at the back of the distal actuation mechanism, to the curved tube following previous work on similar designs [17]. The mechanism’s main components are a leadscrew and square shaft of equal length positioned parallel to one another [Fig. 3(b) and (c)]. The shafts are held in place but allowed to freely rotate along their axis through bearings at either end. A carriage holds the tube suspended by two bearings at either side of the gear (See Fig. 3). The rotation component of the carriage features a gear with a square profile cut through the centre. This allows the square shaft to translate through the profile but fixes the orientation of the gear to the square shaft and the gear is used to drive the tube gear. The translation component of the carriage consists of a lead screw nut fixed to the carriage concentric to the lead screw and it uses the square shaft as the linear guide. A diagram of the carriage is shown in Fig. 3(b). While the mechanism housing holds the lead screw and square shaft, the motors are not fixed to the mechanism. Instead, the motors are fixed to the mounting plate on the proximal manipulator. The motors can then be joined to the mechanism through a hexagonal coupling to be slid into the housing and fixed with a single machine screw. The use of the motor couples allows the mechanism to be motor agnostic. By positioning the motors on the mounting plate and adjacent to the proximal arm, the device can be easily adapted during a procedure either to introduce additional actuation capabilities or imaging functionality.

The majority of components constructing the mechanism housing, carriage and mounting plate were 3D printed using Polylactic acid (PLA), (RS Components, GB) on an Ultimaker 2 (Ultimaker B.V., NL). The tube couples, inner and instrument shaft, were printed using tough resin on a Formlabs 1+ (Formlabs Inc., USA) and the camera housing was printed using a Connex 500 (Stratasys Ltd, US) in Vero Clear material. The linear bearing to provide the interface between the carriage gear and square shaft is printed on the Ultimaker using iglide Tribo-Filament (Igus, DE) which is a wear resistant filament. Actuation is delivered by Dynamixel MX-28 motors (Robotis Inc., US) which drive the mechanism. Distally, the instrument was assembled with a steel outer tube with an inner and outer diameter of 1.8 mm and 2.45 mm respectively and a length of 150 mm, and a nitinol inner tube, 1.4 mm and 1.59 mm in diameter. These specifications allowed a good interface between the tube and maintained a diameter below 3.0 mm with integration to the camera discussed at the end of this section. The nitinol tube was curved following conventional processes [18], where the tube is constrained to the desired shape in a heat-resistant jig then placed in a box furnace at 550° for 20 minutes. The tube was set to a bending radius of 30 mm, to reduce the strain on the imaging components, and arc angle of 80° (Arc length, 41.89 mm), with a straight section, 7 mm long at the tip to join the instruments, and a transmission length of 170 mm. A Naneye Stereo camera (AWAIBA Lda, PT) was integrated into the tip of the inner tube to provide a temporary imaging solution (Fig. 3(a)). The solution is temporary because it effectively occupies the entire working channel leaving no flexibility for including a light source or a therapeutic laser. The Naneye has a profile of 2.2 mm × 1.0 mm and bounding diameter of 2.42 mm and the tubes have an inner diameter of 1.4 mm, an interface between them was made to fix the camera in place. The interface features a rectangular channel 2.4 mm wide for the camera and a connector with a diameter of 1.35 mm to fix it to the inner tube.

## III System Control Design

### A Distal Manipulation: Concentric Tube Robot

**Fig. 4:**
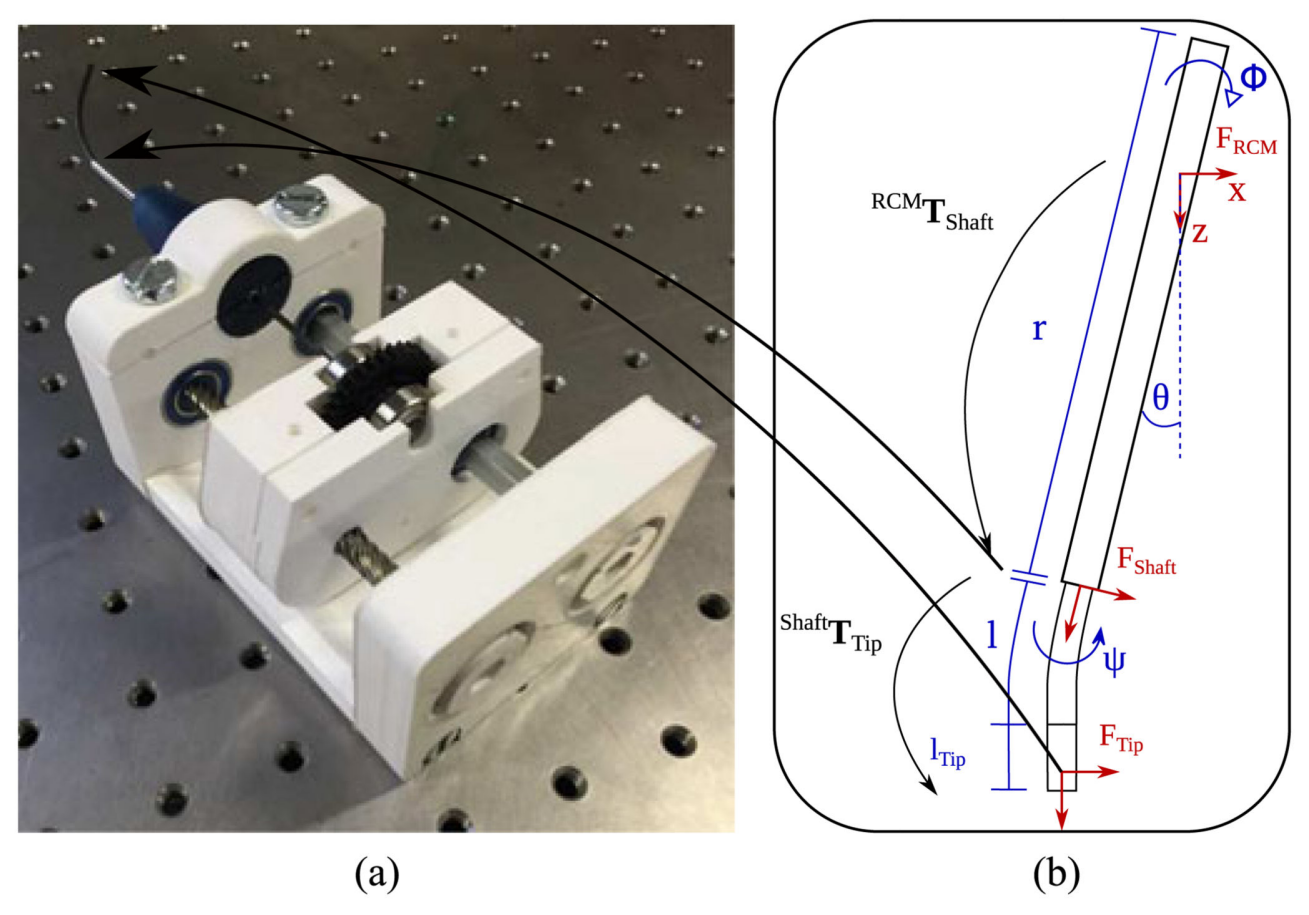
(a) Image of the assembled distal actuation mechanism; (b) Diagram showing the coordinate frames (F) and transformations (T) from the RCM to the tip of the instrument with each kinematic variable.

The principle of our distal mechanism relies on the bending stiffness of the outer straight tube to be significantly larger than the stiffness of the inner tube. This causes the shape of the inner tube to conform to the shape of the outer tube when overlapping. This allows the overall shape of the instrument to be modelled as the shape of the outer tube and followed by the shape of the inner tube with the length extruded from the outer tube. With the inner tube having the fixed parameters, *l_ArcLength_*, the length of the curved section of the tube; *l_TipLength_*, the length of the straight section at the tip extended from the outer tube; and *k*, the curvature of the arc equivalent to the inverse of the bending radius. While the controllable parameters, length extended from outer tube, *p* and bending plane given by the orientation of the tube, *ψ* (Fig. 4). This can be represented as:

(1) $$l_{\text{Tip}}=\{\begin{array}{ll} p, & \text{if}\;p\leq l_{TipLength}\\ l_{TipLength}, & \text{otherwise} \end{array}$$

(2) $$l=\{\begin{array}{ll} 0, & \text{if}\;p\leq l_{TipLength}\\ p-l_{TipLength,} & \text{otherwise} \end{array}$$

(3) $${}^{\text{Shaft}}{\text{T}}_{\psi }=[\begin{array}{cccc} \text{cos}(\psi ) & -\text{sin}(\psi ) & 0 & 0\\ \text{sin}(\psi ) & \text{cos}(\psi ) & 0 & 0\\ 0 & 0 & 1 & 0\\ 0 & 0 & 0 & 1 \end{array}]$$

(4) $${}^{\psi }{\text{T}}_{\text{cur}}=\left[\begin{array}{cccc} \text{cos}(kl) & 0 & \text{sin}(kl) & \frac{\left(\text{cos}(kl)-1\right)}{k}\\ 0 & 1 & 0 & 0\\ -\text{sin}(kl) & 0 & \text{cos}(kl) & \frac{\text{sin}(kl)}{k}\\ 0 & 0 & 0 & 1 \end{array}\right]$$

(5) $${}^{\text{cur}}{\text{T}}_{\text{tip}}=\left[\begin{array}{cccc} 1 & 0 & 0 & 0\\ 0 & 1 & 0 & 0\\ 0 & 0 & 1 & l_{Tip}\\ 0 & 0 & 0 & 1 \end{array}\right]$$

(6) $${}^{\text{Shaft}}{\text{T}}_{\text{Tip}}={}^{\text{Shaft}}\;{\text{T}}_{\psi }\;{}^{\psi }{\text{T}}_{\text{cur}}{}^{\text{cur}}{\text{T}}_{\text{Tip}}$$

Where bold capital letters are used to represent 4 x 4 transformation matrices. and each kinematic variable is limited as follows *ψ* ∈ [−π, π], *p* ∈ [0, *l_ArcLength_* + *l_TipLength_*]. The frames declared are defined as follows: Shaft is the end of the outer tube, *ψ* is used to represent the orientation of the inner tube according to the rotation of the actuator, cur represents the end of the curved section of the inner tube, and Tip is the end of the inner tube.

### B Proximal Manipulation

An articulated arm (KUKA LBR iiwa 7 R800, KUKA Gmbh, DE) is used for the proximal manipulation of the instrument. The arm is a 7DOF robot with torque sensors on each joint. As a minimally invasive procedure, FLP requires the instruments used to be constrained to a RCM due to the small incisions used to access the inner anatomy through a port. An RCM constrains the motion to 4DOF, translation, *r*, and rotations along the instrument shaft (Z axis), and rotations of the X and Y axes. However, the z axis rotation is restricted as the distal actuation mechanism provides continuous rotation along the z axis while the proximal manipulator has a limited range of motion and velocity. From the RCM, the transform of the tip of the instrument shaft, in this case the outer tube, can be shownas:

(7) $${}^{\text{RCM}}{\text{T}}_{\theta }=\left[\begin{array}{cccc} \text{cos}(\theta ) & -\text{sin}(\theta ) & 0 & 0\\ \text{sin}(\theta ) & \text{cos}(\theta ) & 0 & 0\\ 0 & 0 & 1 & 0\\ 0 & 0 & 0 & 1 \end{array}\right]$$

(8) $${}^{\theta }{\text{T}}_{\phi }=\left[\begin{array}{cccc} \text{cos}(\phi ) & 0 & \text{sin}(\phi ) & 0\\ 0 & 1 & 0 & 0\\ -\text{sin}(\phi ) & 0 & \text{cos}(\phi ) & 0\\ 0 & 0 & 0 & 1 \end{array}\right]$$

(9) $${}^{\phi }{\text{T}}_{\text{Shaft}}=\left[\begin{array}{cccc} 1 & 0 & 0 & 0\\ 0 & 1 & 0 & 0\\ 0 & 0 & 1 & r\\ 0 & 0 & 0 & 1 \end{array}\right]$$

(10) $${}^{\text{RCM}}{\text{T}}_{\text{Shaft}}={}^{\text{RCM}}{\text{T}}_{\theta }\;{}^{\theta }{\text{T}}_{\phi }\;{}^{\phi }{\text{T}}_{\text{Shaft}}$$

Where each kinematic variable is limited as: *θ* ∈ [*−π, π*], *ϕ* ∈ [0, *π*] and *r* ∈ [0, *l_OuterTube_*] (with *l_OuterTube_* being the length of the outer tube). As the instrument shaft (Outer Tube) is rigidly fixed to the kuka, the transform between **T**_*Flange*_, given by the KUKA application programming interface (API), and **T**_*Shaft*_ is found by moving the proximal manipulatorabout a series of poses keeping the tip of the shaft stationary, giving the position, while the orientation can be found by aligning the shaft with the world axis. **T**_*RCM*_ is then set with respect to the shaft coordinate system at the start of the procedure.

### C Control Implementation

The control of the instrument is separated to each mechanism, where the cartesian position of the instrument shaft relative to the RCM is set explicitly in the path planning by the surgeon, while the orientation of the tube is set to maintain a constant orientation. The position (x, y, z) of the tip of the outer tube can be explicitly set with respect to the RCM through:

(11) $$r=\sqrt{x^{2}+y^{2}+z^{2}}$$

(12) $$\theta =\text{arctan}(\frac{y}{x})$$

(13) $$\phi =\text{arccos}\left(\frac{z}{r}\right)$$

In controlling the instrument shaft, the position of the tip can be constrained to remain parallel to the Z axis of the RCM. This constraint may be resolved by calculating the orientation of the tube, *ϕ*, that aligns the bending plane of the tube perpendicular to the desired imaging plane; and secondly, length of the tube extended, *l*, must be found to achieve the perpendicular constraint. The orientation of the tube can be found by finding the difference between **T**_*RCM*_ and **T**_*Shaft*_ around the Z axis. While the length can be found by finding the angle between the RCM, the Z axis and the instrument shaft axis, *θ*.

The control system is implemented to run on the Sunrise Cabinet (KUKA) running Windows embedded Operating System (OS) (Microsoft Corp., US) and VxWorks (Wind River Systems Inc., US) providing a soft real-time system. The KUKA is controlled through the DirectServo API, a Java library running on the Windows operating system communicating with the VxWorks OS. The DirectServo API allows the robot’s flange to be controlled in Cartesian space. The entire kinematic chain is implemented in Java, while joint control for the concentric tube mechanism is implemented using the Dynamixel SDK in C++ and integrated with the controller through a Java wrapper.

## IV Experiments and Results

### A Experiments Comparing to EM Tracking

**Fig. 5:**
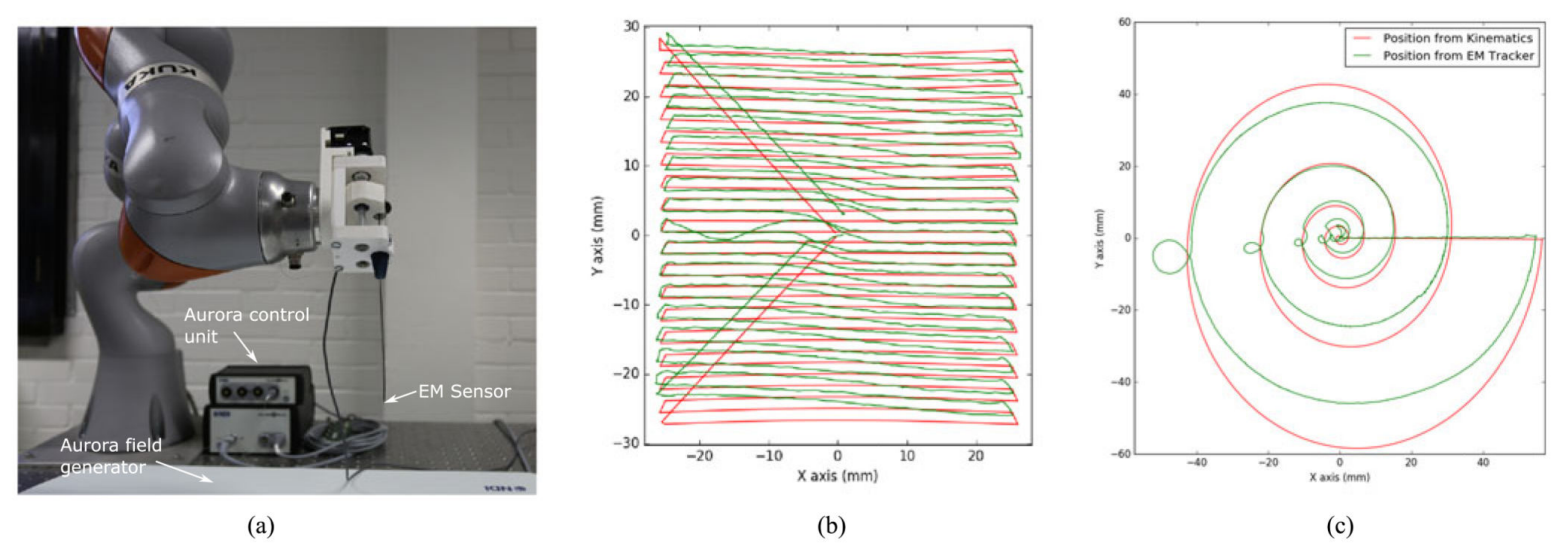
(a) Image of the assembly instrument with EM sensor integrated at the tip; Scanning trajectories from the kinematic data and EM tracker projected to the XY plane as the z position of the shaft remains constant throughout the scan; (b) Raster scan with an area (50 *×* 50) mm^2^ with 1.5 mm steps along the y axis; (c) Spiral scan with a maximum radius of 50 mm and 5 revolutions.

To validate the motion capabilities of the developed mechanism, an electromagnetic (EM) tracker (NDI Aurora, Northern Digital Inc., CA) was passed along the concentric tube and used to compare the motion of the forward kinematics to the EM measurements. The EM probe used was the 6DOF micro-probe which has a diameter of 0.8 mm and length of 9 mm. The probe was fixed using a small printed ring to retain concentricity fabricated using a Connex 500. The RMS accuracy of the tracker is specified as 0.8 mm and 0.7°. The experiment was performed on an optical table (Nexus, Thorlabs Inc., US) with the proximal manipulator, EM field generator, and EM control unit as shown in Fig 5(a). The transformation between EM tracker coordinates and the RCM frame was found by using an adaptation of the hand eye calibration method described in [19] with 30 synchronised poses.

The trajectory of the tip was assessed using the EM tracker and commanding the instrument to follow a series of open-loop scanning trajectories while the tip is constrained to remain perpendicular to the XY plane. Two types of scanning trajectories were followed: spiral scans and raster scans. The spiral trajectory to be applied to the shaft on a XY plane was defined by: number of complete revolutions, c and final radius, R, while each position is generated from iterating along i within it’s limits and the density of the path is determined by the number of i values used.

(14) $$a=i^{\frac{3}{2}}R$$

(15) $$x=a\text{cos}\left(\sqrt{i{\left(2\pi \;c\right)}^{2}}\right),\;y=a\text{sin}\left(\sqrt{i{\left(2\pi \;c\right)}^{2}}\right)$$

(16) wherei∈[0,1]

The raster trajectory was defined by an area to scan and the step along each axis, the scan area was centred to the initial position. The instrument trajectories from the kinematics data and EM tracking data were aligned and due to the difference between the sampling frequencies of each device each pose from the em tracker was matched to a kinematic pose through a distance criterion. Once matched the distance between the points was calculated and the orientation difference was determined by finding the euler angles in ZYZ convention then calculating the difference of the rotation along the Y axis. The kinematic and tracked paths from a raster and spiral scan are shown overlayed in Fig. 5(b) and (c) respectively. For each experiment the RCM was placed 100 mm above the Shaft frame. The raster scan (Fig. 5(b)) had an area of (50 × 50) mm^2^ with 1.5 mm steps along the y axis, the average error between the kinematic position and tracked position was 0.77 mm while the average orientation error was 10.17°. In comparison, the spiral scan had a maximum radius of 50 mm and 5 revolutions, the average translation error is 3.10 mm and the average angular difference is 22.31° as shown in Table I. The repeatability of the scans was assessed though tracking 5 identical trajectories, matching the poses along each trajectory to a reference then finding the average distance to the reference trajectory. The repeatability of the path was 0.77 mm while the combined average translation error was 3.50 mm.

### B Placental Scanning

**Fig. 6:**
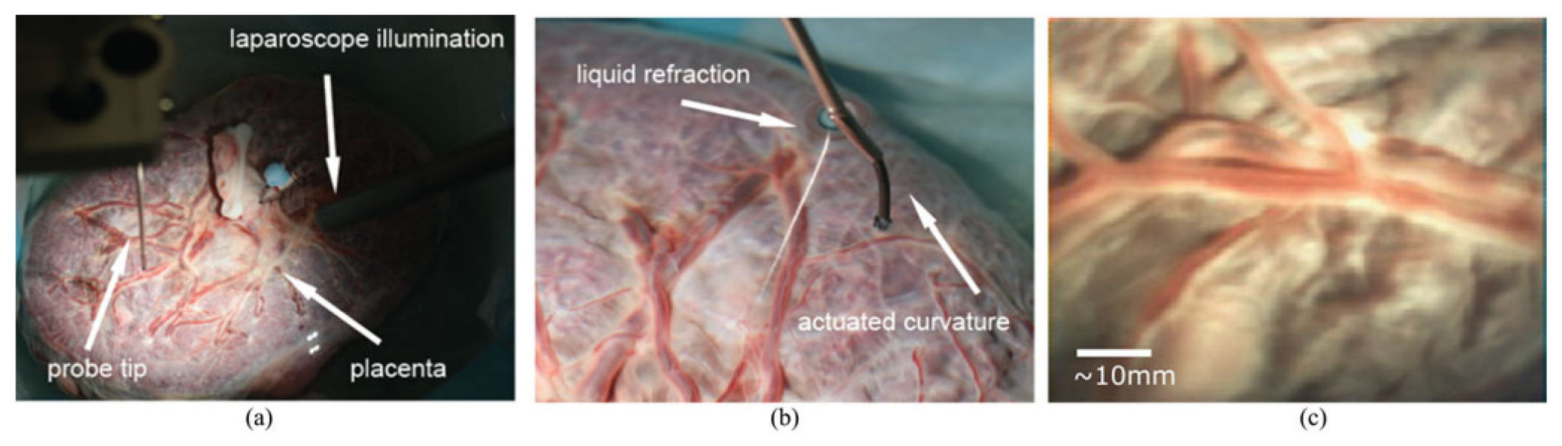
(a) Image of the placenta imaging setup, showing the position of the probe and laparoscope relative to the placenta; (b) Image of the instrument partially submerged in water with the tip extended to view the placenta along the normal to the placenta surface; (c) *ex vivo* imaging of human term placenta.

**Fig. 7:**
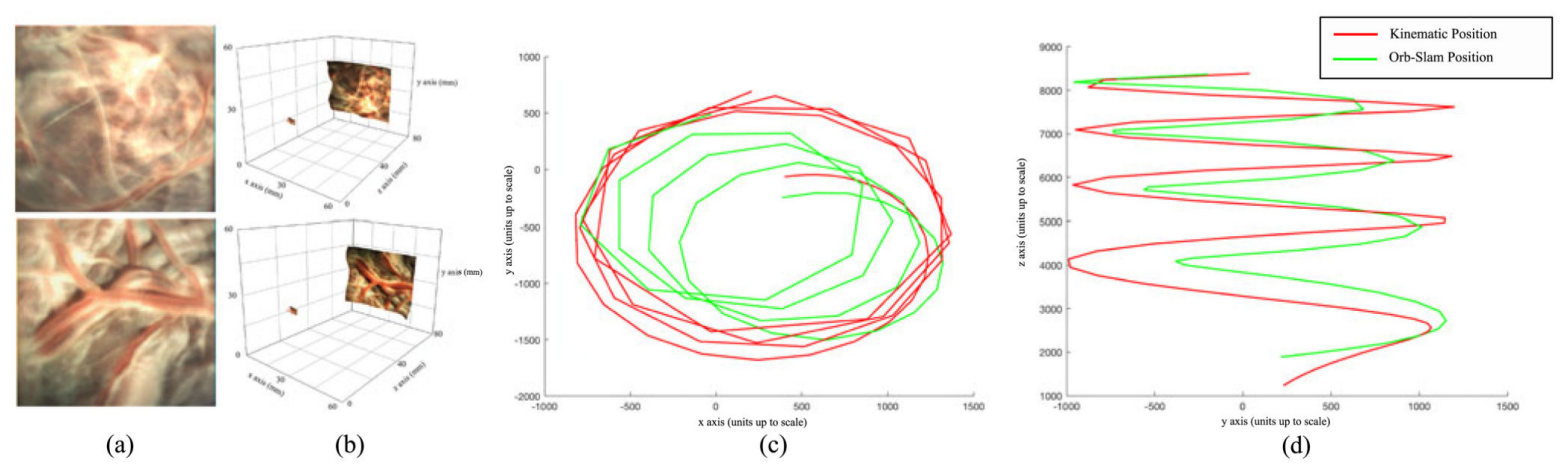
(a) Image from the left channel of the Naneye Stereo camera (AWAIBA Lda, PT) of a human *ex vivo* placenta; (b) 3D rendition of the stereo reconstruction; (c) Trajectory of ORB-SLAM and aligned kinematics in the ORB-SLAM coordinate system: Projected to the XY plane; (d) The trajectory from (c) but projected on the YZ plane.

**Fig. 8:**
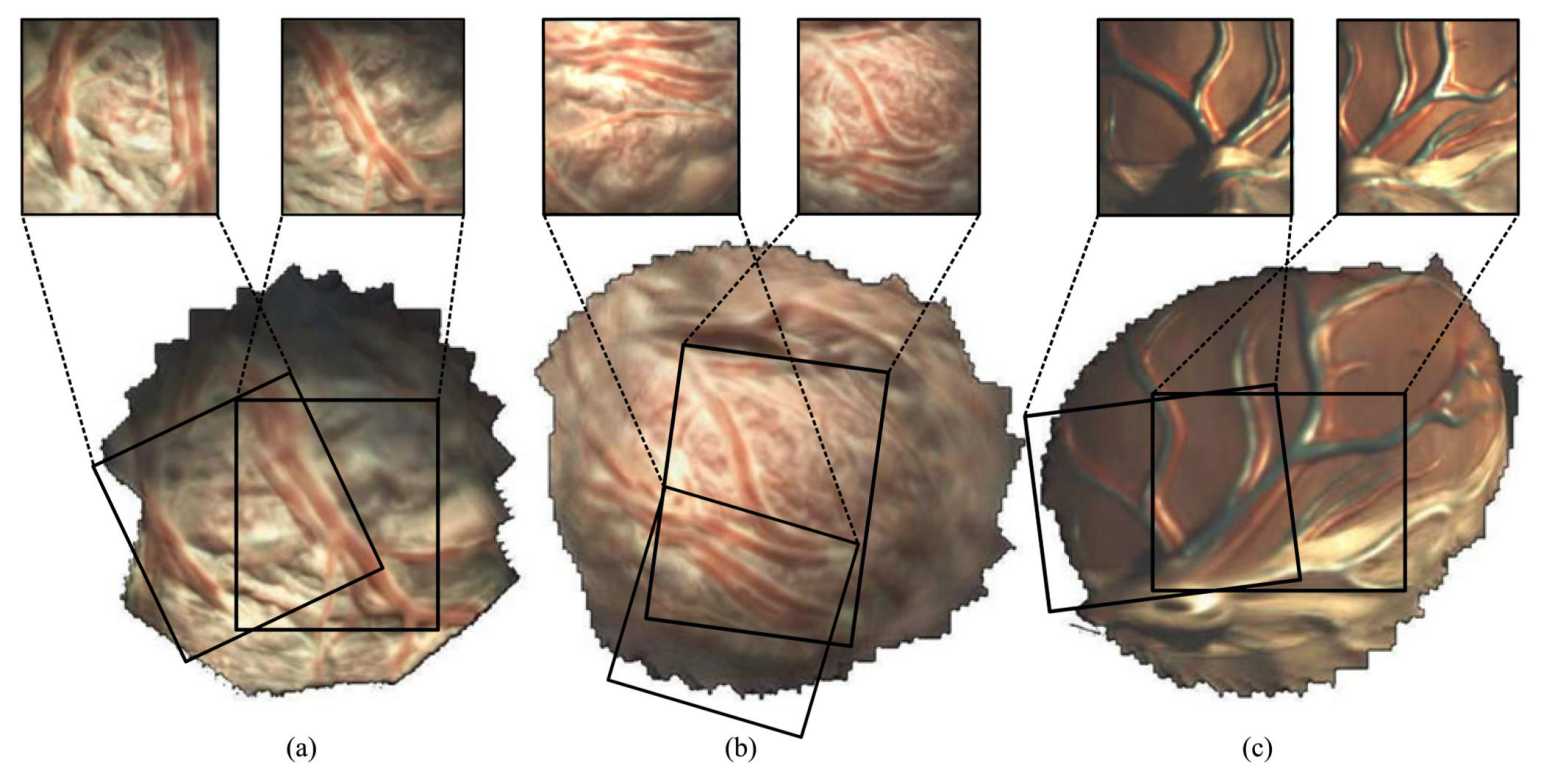
Mosaics formed from the scanning trajectories; (a) Raster scan of the human *ex vivo* placenta, 30 mm *×* 30 mm; (b) Spiral scan of the human *ex vivo* placenta, radius of ≈ 20 mm; (c) Spiral scan of a model placenta, radius of ≈ 20 mm.

To experiment in more realistic conditions and aim towards demonstrating the potential clinical use of the reported instrument, a term (approximately 40 weeks gestation age) human placenta was collected following a caesarean section delivery and after obtaining a written informed consent from the mother at University College London Hospital (UCLH). The Joint UCL/UCLH Committees on the Ethics of Human Research approved the study (ethical approval UCL 08/H0714/87). The placenta was transported to our laboratory and immersed in water within a container. The instrument was immersed in the water and introduced above the placenta as shown in Fig 6(a). Additionally a stereo laparoscope was positioned alongside the instrument to illuminate the workspace as the instrument does not currently have an integrated lightsource. The immersed placenta was imaged a number of times using both raster scans and spiral motion patters acquiring stereoscopic data, the position of the instrument relative to the placenta can be seen in Fig 6(b). The camera acquires synchronised images at 27 frames per second (fps) with a resolution of 250 × 250 in each camera, an image from one of the cameras can be seen in Fig 6(c). To demonstrate the potential information that can be derived from the scans of the placenta, stereoscopic reconstruction of the placenta was undertaken using the method described in [20] and shown in Fig. 7(a) and (b). In addition, the camera trajectory was reconstructed using ORB-SLAM [21] and aligned with the instrument kinematics, however due to the fast “wind back” motions during the spiral scan, the video and kinematics were sub sampled to 60 keyframes with the path shown in Fig. 7(c) and (d). A mosaic using the method described in [22], [23] was constructed of three scanned areas, raster scan and spiral scan of a real placenta, and a spiral scan of a model placenta for Fig. 8(a)–(c) respectively. The mosaics provide an increased field of view of the placenta and crucially the surface vasculature.

## V Discussion

FLP is challenging due to the small field-of-view, which inhibits localisation and makes it difficult to be certain that all vessels are ablated. Intra-operative motion of the fetuses and physiological signals from the mother make it difficult to maintain a measured energy delivery and ensure consistent therapeutic delivery. The instrument we have developed improves the dexterity of the fetoscope by providing DOF at the tip and enhances the ergonomics of the system by an RCM constrained arm. Our prototype was fabricated using mostly 3D printed components. The current design allows the tubes to be easily swapped and the joint workspace to be modified as the system evolves. The layout of the mechanism and the interface with the actuators, groups the non-sterilisable objects (actuators and proximal manipulator) together. This design decision was made bearing in mind clinical translation to allow a single sterile sheet to be used with two coupling adaptors to transmit the motion through the sterile barrier. The RCM is currently placed manually by the operator, however the intention is for the RCM to be placed at the amniotic sac entry point, minimising forces applied to the membranes, this may require sensor guidance for accurate results.

Our current model for the distal manipulation assumes that the outer tube is rigid, there is no torsion along the nitinol tube and that the straight tip of the tube is extended from the outer tube along the outer tube axis. This model has the advantage of being quick to solve but can be improved in accuracy. This is reflected in the trajectory comparisons, shown in Fig. 5 where the pose accuracy of the instrument was shown to be 3.10 *±* 11.91 mm and 22.31 *±* 5.93° for the spiral scans and 0.77 *±* 0.25 mm and 10.17 *±* 0.59° for the spiral scans. This disparity in accuracy information is likely due to the comparison of unsynchronised trajectories, and the individual poses being incorrectly matched. Synchronisation of the instrument controller and tracker could allow poses to be directly measured against one another. However, the repeatability of the trajectories was shown to be 0.77 mm, we believe that learning approaches can help us to better parameterize the control of the concentric tube while maintaining computational performance.

In our experiments, we show the combination of the proximal motion controlled by the manipulator constrained to a RCM and the distal motion from the concentric tube. We performed two scanning motions that have regular structure for analysis: raster and spiral. Spiral motions are often applied in visual scanning mechanisms [24], [25], they require the imaging component to be capable of continuous rotation or to be “wound back” to an initial position. Our current system resolves this by limiting the rotation to a single revolution. While this prevents wind up, it also affects the trajectory of the instrument during scans especially spirals as the rotation has to reset during the scan interrupting the commanded trajectory. This can be seen in Fig. 5(c) along the left side of each revolution. In comparison the raster scan has an offset at the peripheral, however, it does not have the sudden “wind back” motions. We plan to explore the links between kinematics and imaging more in future studies because stereoscopic reconstruction has the potential to provide additional information during the procedure, for example the distance from the camera to the vessels and vessel size. The mosaics presented show the potential to increase the field of view intra operatively, however, they do not currently use the kinematic data in the reconstruction process which similar to other tracking systems would allow larger and more accurate mosaics.

## VI Conclusions

We have developed a new concentric tube robot designed for fetoscopic procedures where the instrument needs to be small with limited DOF at the tip. Our mechanism has a small diameter of 2.4 mm in order to minimise access trauma which is crucial in fetoscopy. While this is the first version of our robot and hence aspects are still preliminary, the architecture has the advantage of being extensible. The control system we implemented was developed to improve fetoscopic interventions by providing a stable view of the placenta at a constant orientation. This capability will facilitate surgical assist in the delivery laser photocoagulation of placental vasculature for the treatment of TTTS. Our future work, will focus on integration of a therapeutic laser and light source at the tip to understand the practical use of our system as well as developing a control scheme to allow comanipulation while minimising the forces at the entry point by adapting the position of the RCM according to external forces such as those from patient motion.

## Figures and Tables

**Table I:** Metrics on Trajectories, Showing the Mean and Standard Deviation

	Translation Error (mm)	Angular Error (deg)
**Spiral Scan**	3.10 ± 11.91	22.31 ± 5.83
**Raster Scan**	0.77 ± 0.25	10.17 ± 0.59
